# Supplementary material for: A fault-intrusion-tolerant system and deadline-aware algorithm for scheduling scientific workflow in the cloud
Source: PeerJ Comput Sci. 2021 Nov 2;7:e747. doi: 10.7717/peerj-cs.747 (PMC8576554; doi:10.7717/peerj-cs.747)
Supplement: Supplemental Information 6 [file peerj-cs-07-747-s006.zip › Myproject1.1/docs/org/cloudbus/cloudsim/core/CloudSim.html]

CloudSim


JavaScript is disabled on your browser.


- Overview
- Package
- Class
- Use
- Tree
- Deprecated
- Index
- Help

- Prev Class
- Next Class

- Frames
- No Frames

- All Classes

- Summary:
- Nested |
- Field |
- Constr |
- Method

- Detail:
- Field |
- Constr |
- Method


org.cloudbus.cloudsim.core

## Class CloudSim

- java.lang.Object
- - org.cloudbus.cloudsim.core.CloudSim

- ---

    

  ```
  public class CloudSim
  extends java.lang.Object
  ```

  This class extends the CloudSimCore to enable network simulation in CloudSim. Also, it disables
  all the network models from CloudSim, to provide a simpler simulation of networking. In the
  network model used by CloudSim, a topology file written in BRITE format is used to describe the
  network. Later, nodes in such file are mapped to CloudSim entities. Delay calculated from the
  BRITE model are added to the messages send through CloudSim. Messages using the old model are
  converted to the apropriate methods with the correct parameters.

  Since:
  :   CloudSim Toolkit 1.0

- - ### Field Summary

    Fields

    | Modifier and Type | Field and Description |
    | --- | --- |
    | `protected static DeferredQueue` | `deferred` The deferred event queue. |
    | `protected static FutureQueue` | `future` The future event queue. |
    | `static PredicateAny` | `SIM_ANY` A standard predicate that matches any event. |
    | `static PredicateNone` | `SIM_NONE` A standard predicate that does not match any events. |
  - ### Constructor Summary

    Constructors

    Constructor and Description

    `CloudSim()`
  - ### Method Summary

    Methods

    | Modifier and Type | Method and Description |
    | --- | --- |
    | `static void` | `abruptallyTerminate()` Abruptally terminate. |
    | `static void` | `addEntity(SimEntity e)` Add a new entity to the simulation. |
    | `protected static void` | `addEntityDynamically(SimEntity e)` Internal method used to add a new entity to the simulation when the simulation is running. |
    | `static SimEvent` | `cancel(int src, Predicate p)` Removes an event from the event queue. |
    | `static boolean` | `cancelAll(int src, Predicate p)` Removes all events that match a given predicate from the future event queue returns true if at least one event has been cancelled; false otherwise. |
    | `static double` | `clock()` Get the current simulation time. |
    | `static SimEvent` | `findFirstDeferred(int src, Predicate p)` Find first deferred event matching a predicate. |
    | `static void` | `finishSimulation()` Internal method that allows the entities to terminate. |
    | `static int` | `getCloudInfoServiceEntityId()` Gets the entity ID of CloudInformationService. |
    | `static java.util.List<java.lang.Integer>` | `getCloudResourceList()` Sends a request to Cloud Information Service (GIS) entity to get the list of all Cloud hostList. |
    | `static SimEntity` | `getEntity(int id)` Get the entity with a given id. |
    | `static SimEntity` | `getEntity(java.lang.String name)` Get the entity with a given name. |
    | `static int` | `getEntityId(java.lang.String name)` Get the id of an entity with a given name. |
    | `static java.util.List<SimEntity>` | `getEntityList()` Returns a list of entities created for the simulation. |
    | `static java.lang.String` | `getEntityName(int entityID)` Gets name of the entity given its entity ID. |
    | `static java.lang.String` | `getEntityName(java.lang.Integer entityID)` Gets name of the entity given its entity ID. |
    | `static double` | `getMinTimeBetweenEvents()` Returns the minimum time between events. |
    | `static int` | `getNumEntities()` Get the current number of entities in the simulation. |
    | `static java.util.Calendar` | `getSimulationCalendar()` Gets a new copy of initial simulation Calendar. |
    | `static void` | `hold(int src, long delay)` Used to hold an entity for some time. |
    | `static void` | `init(int numUser, java.util.Calendar cal, boolean traceFlag)` Initialises CloudSim parameters. |
    | `static void` | `init(int numUser, java.util.Calendar cal, boolean traceFlag, double periodBetweenEvents)` Initialises CloudSim parameters. |
    | `protected static void` | `initialize()` Initialise the simulation for stand alone simulations. |
    | `static boolean` | `isPaused()` Checks if is paused. |
    | `static void` | `pause(int src, double delay)` Used to pause an entity for some time. |
    | `static boolean` | `pauseSimulation()` This method is called if one wants to pause the simulation. |
    | `static boolean` | `pauseSimulation(long time)` This method is called if one wants to pause the simulation at a given time. |
    | `static boolean` | `resumeSimulation()` This method is called if one wants to resume the simulation that has previously been paused. |
    | `static double` | `run()` Start the simulation running. |
    | `static boolean` | `runClockTick()` Internal method used to run one tick of the simulation. |
    | `static boolean` | `running()` Check if the simulation is still running. |
    | `static void` | `runStart()` Internal method used to start the simulation. |
    | `static void` | `runStop()` Internal method used to stop the simulation. |
    | `static SimEvent` | `select(int src, Predicate p)` Selects an event matching a predicate. |
    | `static void` | `send(int src, int dest, double delay, int tag, java.lang.Object data)` Used to send an event from one entity to another. |
    | `static void` | `sendFirst(int src, int dest, double delay, int tag, java.lang.Object data)` Used to send an event from one entity to another, with priority in the queue. |
    | `static double` | `startSimulation()` Starts the execution of CloudSim simulation. |
    | `static void` | `stopSimulation()` Stops Cloud Simulation (based on `Simulation#runStop()`). |
    | `static boolean` | `terminateSimulation()` This method is called if one wants to terminate the simulation. |
    | `static boolean` | `terminateSimulation(double time)` This method is called if one wants to terminate the simulation at a given time. |
    | `static void` | `wait(int src, Predicate p)` Sets an entity's state to be waiting. |
    | `static int` | `waiting(int d, Predicate p)` Checks if events for a specific entity are present in the deferred event queue. |

    - ### Methods inherited from class java.lang.Object

      `clone, equals, finalize, getClass, hashCode, notify, notifyAll, toString, wait, wait, wait`

- - ### Field Detail


    - #### future

      ```
      protected static FutureQueue future
      ```

      The future event queue.


    - #### deferred

      ```
      protected static DeferredQueue deferred
      ```

      The deferred event queue.


    - #### SIM\_ANY

      ```
      public static final PredicateAny SIM_ANY
      ```

      A standard predicate that matches any event.


    - #### SIM\_NONE

      ```
      public static final PredicateNone SIM_NONE
      ```

      A standard predicate that does not match any events.
  - ### Constructor Detail


    - #### CloudSim

      ```
      public CloudSim()
      ```
  - ### Method Detail


    - #### init

      ```
      public static void init(int numUser,
              java.util.Calendar cal,
              boolean traceFlag)
      ```

      Initialises CloudSim parameters. This method should be called before creating any entities.

      Inside this method, it will create the following CloudSim entities:
      - CloudInformationService.- CloudSimShutdown

      Parameters:
      :   `numUser` - the number of User Entities created. This parameters indicates that
          `gridsim.CloudSimShutdown` first waits for all user entities's
          END\_OF\_SIMULATION signal before issuing terminate signal to other entities
      :   `cal` - starting time for this simulation. If it is null, then the time will be
          taken from Calendar.getInstance()
      :   `traceFlag` - true if CloudSim trace need to be written

      See Also:
      :   `gridsim.CloudSimShutdown`,
          `CloudInformationService.CloudInformationService`


    - #### init

      ```
      public static void init(int numUser,
              java.util.Calendar cal,
              boolean traceFlag,
              double periodBetweenEvents)
      ```

      Initialises CloudSim parameters. This method should be called before creating any entities.

      Inside this method, it will create the following CloudSim entities:
      - CloudInformationService.- CloudSimShutdown

      Parameters:
      :   `numUser` - the number of User Entities created. This parameters indicates that
          `gridsim.CloudSimShutdown` first waits for all user entities's
          END\_OF\_SIMULATION signal before issuing terminate signal to other entities
      :   `cal` - starting time for this simulation. If it is null, then the time will be
          taken from Calendar.getInstance()
      :   `traceFlag` - true if CloudSim trace need to be written
      :   `periodBetweenEvents` - - the minimal period between events. Events within shorter periods
          after the last event are discarded.

      See Also:
      :   `gridsim.CloudSimShutdown`,
          `CloudInformationService.CloudInformationService`


    - #### startSimulation

      ```
      public static double startSimulation()
                                    throws java.lang.NullPointerException
      ```

      Starts the execution of CloudSim simulation. It waits for complete execution of all entities,
      i.e. until all entities threads reach non-RUNNABLE state or there are no more events in the
      future event queue.

      **Note**: This method should be called after all the entities have been setup and added.

      Returns:
      :   the double

      Throws:
      :   `java.lang.NullPointerException` - This happens when creating this entity before initialising
          CloudSim package or this entity name is null or empty.

      See Also:
      :   `gridsim.CloudSim#init(int, Calendar, boolean)`


    - #### stopSimulation

      ```
      public static void stopSimulation()
                                 throws java.lang.NullPointerException
      ```

      Stops Cloud Simulation (based on `Simulation#runStop()`). This should be only called if
      any of the user defined entities **explicitly** want to terminate simulation during
      execution.

      Throws:
      :   `java.lang.NullPointerException` - This happens when creating this entity before initialising
          CloudSim package or this entity name is null or empty

      See Also:
      :   `gridsim.CloudSim#init(int, Calendar, boolean)`,
          `Simulation#runStop()`


    - #### terminateSimulation

      ```
      public static boolean terminateSimulation()
      ```

      This method is called if one wants to terminate the simulation.

      Returns:
      :   true, if successful; false otherwise.


    - #### terminateSimulation

      ```
      public static boolean terminateSimulation(double time)
      ```

      This method is called if one wants to terminate the simulation at a given time.

      Parameters:
      :   `time` - the time at which the simulation has to be terminated

      Returns:
      :   true, if successful otherwise.


    - #### getMinTimeBetweenEvents

      ```
      public static double getMinTimeBetweenEvents()
      ```

      Returns the minimum time between events. Events within shorter periods after the last event are discarded.

      Returns:
      :   the minimum time between events.


    - #### getSimulationCalendar

      ```
      public static java.util.Calendar getSimulationCalendar()
      ```

      Gets a new copy of initial simulation Calendar.

      Returns:
      :   a new copy of Calendar object or if CloudSim hasn't been initialized

      See Also:
      :   `gridsim.CloudSim#init(int, Calendar, boolean, String[], String[], String)`,
          `gridsim.CloudSim#init(int, Calendar, boolean)`


    - #### getCloudInfoServiceEntityId

      ```
      public static int getCloudInfoServiceEntityId()
      ```

      Gets the entity ID of CloudInformationService.

      Returns:
      :   the Entity ID or if it is not found


    - #### getCloudResourceList

      ```
      public static java.util.List<java.lang.Integer> getCloudResourceList()
      ```

      Sends a request to Cloud Information Service (GIS) entity to get the list of all Cloud
      hostList.

      Returns:
      :   A List containing CloudResource ID (as an Integer object) or if a CIS entity hasn't
          been created before


    - #### initialize

      ```
      protected static void initialize()
      ```

      Initialise the simulation for stand alone simulations. This function should be called at the
      start of the simulation.


    - #### clock

      ```
      public static double clock()
      ```

      Get the current simulation time.

      Returns:
      :   the simulation time


    - #### getNumEntities

      ```
      public static int getNumEntities()
      ```

      Get the current number of entities in the simulation.

      Returns:
      :   The number of entities


    - #### getEntity

      ```
      public static SimEntity getEntity(int id)
      ```

      Get the entity with a given id.

      Parameters:
      :   `id` - the entity's unique id number

      Returns:
      :   The entity, or if it could not be found


    - #### getEntity

      ```
      public static SimEntity getEntity(java.lang.String name)
      ```

      Get the entity with a given name.

      Parameters:
      :   `name` - The entity's name

      Returns:
      :   The entity


    - #### getEntityId

      ```
      public static int getEntityId(java.lang.String name)
      ```

      Get the id of an entity with a given name.

      Parameters:
      :   `name` - The entity's name

      Returns:
      :   The entity's unique id number


    - #### getEntityName

      ```
      public static java.lang.String getEntityName(int entityID)
      ```

      Gets name of the entity given its entity ID.

      Parameters:
      :   `entityID` - the entity ID

      Returns:
      :   the Entity name or if this object does not have one


    - #### getEntityName

      ```
      public static java.lang.String getEntityName(java.lang.Integer entityID)
      ```

      Gets name of the entity given its entity ID.

      Parameters:
      :   `entityID` - the entity ID

      Returns:
      :   the Entity name or if this object does not have one


    - #### getEntityList

      ```
      public static java.util.List<SimEntity> getEntityList()
      ```

      Returns a list of entities created for the simulation.

      Returns:
      :   the entity iterator


    - #### addEntity

      ```
      public static void addEntity(SimEntity e)
      ```

      Add a new entity to the simulation. This is present for compatibility with existing
      simulations since entities are automatically added to the simulation upon instantiation.

      Parameters:
      :   `e` - The new entity


    - #### addEntityDynamically

      ```
      protected static void addEntityDynamically(SimEntity e)
      ```

      Internal method used to add a new entity to the simulation when the simulation is running. It
      should **not** be called from user simulations.

      Parameters:
      :   `e` - The new entity


    - #### runClockTick

      ```
      public static boolean runClockTick()
      ```

      Internal method used to run one tick of the simulation. This method should **not** be
      called in simulations.

      Returns:
      :   true, if successful otherwise


    - #### runStop

      ```
      public static void runStop()
      ```

      Internal method used to stop the simulation. This method should **not** be used directly.


    - #### hold

      ```
      public static void hold(int src,
              long delay)
      ```

      Used to hold an entity for some time.

      Parameters:
      :   `src` - the src
      :   `delay` - the delay


    - #### pause

      ```
      public static void pause(int src,
               double delay)
      ```

      Used to pause an entity for some time.

      Parameters:
      :   `src` - the src
      :   `delay` - the delay


    - #### send

      ```
      public static void send(int src,
              int dest,
              double delay,
              int tag,
              java.lang.Object data)
      ```

      Used to send an event from one entity to another.

      Parameters:
      :   `src` - the src
      :   `dest` - the dest
      :   `delay` - the delay
      :   `tag` - the tag
      :   `data` - the data


    - #### sendFirst

      ```
      public static void sendFirst(int src,
                   int dest,
                   double delay,
                   int tag,
                   java.lang.Object data)
      ```

      Used to send an event from one entity to another, with priority in the queue.

      Parameters:
      :   `src` - the src
      :   `dest` - the dest
      :   `delay` - the delay
      :   `tag` - the tag
      :   `data` - the data


    - #### wait

      ```
      public static void wait(int src,
              Predicate p)
      ```

      Sets an entity's state to be waiting. The predicate used to wait for an event is now passed
      to Sim\_system. Only events that satisfy the predicate will be passed to the entity. This is
      done to avoid unnecessary context switches.

      Parameters:
      :   `src` - the src
      :   `p` - the p


    - #### waiting

      ```
      public static int waiting(int d,
                Predicate p)
      ```

      Checks if events for a specific entity are present in the deferred event queue.

      Parameters:
      :   `d` - the d
      :   `p` - the p

      Returns:
      :   the int


    - #### select

      ```
      public static SimEvent select(int src,
                    Predicate p)
      ```

      Selects an event matching a predicate.

      Parameters:
      :   `src` - the src
      :   `p` - the p

      Returns:
      :   the sim event


    - #### findFirstDeferred

      ```
      public static SimEvent findFirstDeferred(int src,
                               Predicate p)
      ```

      Find first deferred event matching a predicate.

      Parameters:
      :   `src` - the src
      :   `p` - the p

      Returns:
      :   the sim event


    - #### cancel

      ```
      public static SimEvent cancel(int src,
                    Predicate p)
      ```

      Removes an event from the event queue.

      Parameters:
      :   `src` - the src
      :   `p` - the p

      Returns:
      :   the sim event


    - #### cancelAll

      ```
      public static boolean cancelAll(int src,
                      Predicate p)
      ```

      Removes all events that match a given predicate from the future event queue returns true if
      at least one event has been cancelled; false otherwise.

      Parameters:
      :   `src` - the src
      :   `p` - the p

      Returns:
      :   true, if successful


    - #### runStart

      ```
      public static void runStart()
      ```

      Internal method used to start the simulation. This method should **not** be used by user
      simulations.


    - #### running

      ```
      public static boolean running()
      ```

      Check if the simulation is still running. This method should be used by entities to check if
      they should continue executing.

      Returns:
      :   if the simulation is still running, otherwise


    - #### pauseSimulation

      ```
      public static boolean pauseSimulation()
      ```

      This method is called if one wants to pause the simulation.

      Returns:
      :   true, if successful otherwise.


    - #### pauseSimulation

      ```
      public static boolean pauseSimulation(long time)
      ```

      This method is called if one wants to pause the simulation at a given time.

      Parameters:
      :   `time` - the time at which the simulation has to be paused

      Returns:
      :   true, if successful otherwise.


    - #### resumeSimulation

      ```
      public static boolean resumeSimulation()
      ```

      This method is called if one wants to resume the simulation that has previously been paused.

      Returns:
      :   if the simulation has been restarted or or otherwise.


    - #### run

      ```
      public static double run()
      ```

      Start the simulation running. This should be called after all the entities have been setup
      and added, and their ports linked.

      Returns:
      :   the double last clock value


    - #### finishSimulation

      ```
      public static void finishSimulation()
      ```

      Internal method that allows the entities to terminate. This method should **not** be used
      in user simulations.


    - #### abruptallyTerminate

      ```
      public static void abruptallyTerminate()
      ```

      Abruptally terminate.


    - #### isPaused

      ```
      public static boolean isPaused()
      ```

      Checks if is paused.

      Returns:
      :   true, if is paused


- Overview
- Package
- Class
- Use
- Tree
- Deprecated
- Index
- Help

- Prev Class
- Next Class

- Frames
- No Frames

- All Classes

- Summary:
- Nested |
- Field |
- Constr |
- Method

- Detail:
- Field |
- Constr |
- Method
